# Women Who Have Practiced Midwifery in Modern Times

**Published:** 1861-05

**Authors:** 


**Women who have Practiced Midwifery in Modern Times.**—Following the same guide* as when we spoke of women who have practiced medicine in ancient times, we shall now gossip, for awhile, on the subject of those persons of the same sex who have been practitioners of midwifery in modern times. On the first occasion we could only find, here and there, a reference to midwives, *apropos* of the child-birth of certain queens and princesses. Thus, for instance, we are told by Ammianus Marcellinus, that Eusebia, the wife of Constantius, being jealous of the fecundity of her sister-in-law, the wife of Julian the Apostate, bribed the midwife of the former personage to cause the death of her child by cutting the umbilical cord too short.

During the dark ages, the sciences were neglected and medicine little studied. The names of but few surgeons of those times have reached us, and no allusion even is made to midwives, although it is presumable that as the wants of the sex were always the same, these persons continued to be the chief practitioners in midwifery. There is, however, one name of some note in the thirteenth century, that of Trotula, who wrote a work, entitled "*De Mulieri passionibus ante, in et post Partum.*" We speak of Trotula as the writer of this book, although we are aware that claims of its authorship have been set up by some in favor of Eros, a physician and freedman of Julia, the daughter of Augustus, and by others in advocacy of a physician of the same name who also lived at Salernum, of which the midwife Trotula was a native. Delacroix, author of a biography of celebrated midwives, speaks of the above work in the following terms: "A grammatical and medical examination of this book, even its entire texture, all show the imprint of a female mind. The negligence of style, the odd formulas which it contains, the manner in which things are presented, prove, abundantly, that the author was initiated into a number of little secrets which women only communicate to each other." There is another tract by Trotula, in MS., in the Medicean Library at Florence, under the odd title: '*In utilitatem mulierum, et pro decoratione earum, scilicet de facie et vulva earum.*" The conjunction of parts here specified for decoration is certainly quite unexpected, and it shows that, whatever may have been the restraints on free speech, there certainly were none on free writing, in what have been called the dark ages, provided that polemics were not discussed.

The first of the French midwives of whom we have any precise accounts is Perrette, (*sage-femme ou ventrière jurée.*) She lived in the latter part of the fourteenth and beginning of the fifteenth century. It is not known at what time midwives were sworn into the exercise of their calling; the laws and regulations on this subject are lost, or

* Dr. Rouyer, Etudes Medicales sur l'Ancienne Rome.

cannot yet be found. The most marked incident in the life of Perrette was the accusation against her of witchcraft, in that she procured the dead body of a new-born child for another midwife, in order that ointment, put into the hand of this little body, might be rubbed on the skin of a leprous patient, in the expectation, and indeed assured promise, that the crusts would, in consequence, be detached from the face of this afflicted person. Perrette, after seven weeks imprisonment, was condemned, as well as her associate, Catharine, to the pillory, and to be deprived of her title of midwife, (*ventrière*.) She was subjected to the penalty incurred in the first part of the sentence, but, by a special act of royal clemency, Charles VI., then king, authorized Perrette to continue her vocation. The reasons for this act are recited on the occasion. They are, that, by her fidelity, diligence, and industry, she had acquired the love and favor of many noble women, citizens' wives, and others; and that her calling and office are very necessary to the public good, and that many women, who have great confidence in her knowledge and diligence, call daily for her services. This letter of pardon is dated May 17th, 1408. Perrette, who had been engaged in her vocation for twenty years preceding this date, died in 1411.

In coming down to the sixteenth century we begin to find more details respecting midwives. We learn that at this period they attended in nearly all cases of child-birth, especially those which were easy, and they only called in physicians or surgeons when they encountered any difficulty in the case. It would seem that no measures had been taken to give regular instruction to midwives, and to confer on them the right of practicing. Something we learn on this subject in directing our attention to Louise Bourgeois, one of the most celebrated women of this class. She left behind her several works on the art of midwifery, the chief one of which is entitled "Different Observations on Sterility, Abortion, (*perte defruict*,) Fecundity, Childbirth, and Diseases of Women, and New-born Children," forming an octavo volume. This work was published in 1609. Facing its title-page was a portrait of Queen Maria de Medici, to whom it was dedicated, and one of Louise Bourgeois, beneath which were complimentary verses, written by a certain S. Hacquin. At the foot of the portrait of the midwife we read the words, *aged forty-five years*, a specimen of female frankness not often exhibited, even by a midwife, on so delicate a subject in the eyes of the sex generally. The first chapter of this work has for heading: "How I learned the Art of being a Midwife." Louise Bourgeois, born in Paris in 1563, married a surgeon of Tours, named Boursier. In a short time after their marriage they were ruined by the political troubles, by which France was so sorely disturbed; and Louise was obliged to have recourse to her needle for her support. We shall not attempt to translate, but copy in the old French, the specifications of the various fashions of work in which she had a hand, viz.: "*petit poinct, petit mestier, broderie en jarretières*." She was induced to become a *sage-femme* at the instance of a good woman, who had attended her in her several confinements, and who assured that with her abillty to read and write she would soon reach the highest position in the art of midwifery. After some hesitation, arising chiefly from her dread at holding infants during the act of baptism, as was then the usage for midwives, Louise determined to begin her studies. The first work which she read was that of Ambrose Paré, and her first patient was the wife of their porter. She had read that a woman who had just been delivered must not be let sleep for fear that she would be overcome by debility resulting from the evacuation. Louise, for this purpose, kept talking to the newly-become mother; but on one occasion not receiving any answer, she put the child down on the floor, and hurried to her patient, whom she found in a swoon. From this state she was, however, soon recovered by vinegar and water; but in what manner used does not appear. From attending on the poor, Louise rose to being employed by those in easy circumstances, and now she determined to be regularly received (*jurée*) in the society of the *sages-femmes* of Paris. At a reception of this nature it was necessary that there should be present a physician, two surgeons, and two midwives. The latter were the *dame* Dupuis and the *dame* Peronne. These dames, on learning the profession of the husband of the candidate, refused to receive her, on the ground that, as the wife of a surgeon, she would come to an understanding with the doctors, like cut-purses at a fair, and that their vocation would be at an end. They plagued poor Louise with so many silly speeches and sallies, that a fine child of her own, whom she was suckling at the time, died of ennui. One would suppose that the mother would have been the chief sufferer from this cause. The prediction of her own *sage-femme* was at last verified, and Louise Bourgeois had the honor of being chosen to attend Queen Maria de Medici, wife of Henry IV. She left a work, entitled "*On the Birth of my Lords and Ladies, children of France, with the particulars relating to the same, of a remarkable kind.*" The recital of the first confinement of Maria de Medici is, to use the language of M. Malgaigne, one of the most curious historical pictures; on the score of attraction it may be placed alongside the narrative of the siege of Metz, by Ambrose Paré. Henry IV. has perhaps never been so well portrayed in his inner life, and, as it were, in dishabille. The queen was brought to bed six times in nine years, under the care of Louise Bourgeois, who received on the occasion of the birth of a son five hundred crowns, and on that of a daughter three hundred crowns. After the birth of her sixth child the queen asked for a pension of six hundred crowns for her sage-femme, but Henry IV. would only grant three hundred crowns for this object.

Louise Bourgeois had several children, one of whom, a daughter, became also a midwife; but notwithstanding the great pains taken by her mother to have her thoroughly educated for the exercise of her profession, she never attained distinction in it. Louise's goodness of heart was not spoiled by her peeps at high life and at the splendor of a court. Her advice to her daughter is full of pure ethics and inculcations of kindness to all those who ask for her professional services, no matter how poor they may be. After cautioning against all wrongdoing, she specifies, as more particularly to be shunned, the wickedness of giving drugs to procure abortion. It is not enough, she says, to refuse to teach how this can be brought about, or to withhold a remedy for the purpose; "but you must be on your guard, so as not to be deceived by crafty persons, who may insidiously ask you about the diseases of young females and of married women, whom they allege to be of good character." Can we find anywhere more self-denying charity in the practice of medicine than is inculcated in the following passage, addressed by Louise Bourgeois to her daughter?

"When you are called to a house, inform yourself carefully what kind of people are in it, and whether they are of good repute. Were they the poorest people in the world, attend on them with as much zeal as if you were to receive a large reward; and you will take good care, if you discover them to be victims of poverty, not to take a single farthing from them; for to a poor person a little is a great deal. Give to rather than take from them; God will pay you back with large interest. And give thanks to God that he places this in your power, and has chosen you to serve him among his people."

There were, even at this time, houses in which women were delivered, but which were more frequently receptacles for procuring abortion and for prostitution. Similar dens are, we fear, to be found in our own great cities.

The chief professional work of Louise Bourgeois, that one already cited, contains precepts relative to difficult labors, and to diseases of women and of children. One chapter, on uterine hemorrhages depending on misplaced insertion of the placenta, is worthy of notice. In it we are told that there is a state of things in which labor must be at once brought on, no matter what may be the period of pregnancy, in order to preserve her life. At the end of the work there are several interesting cases; among others, that of a vesical calculus which she succeeded in extracting. She cites also the case of a little girl who had no anus, and in whom the lower part of the rectum was wanting: this gut opened into the vagina at a certain distance from the vulva. No operation was attempted in this case. Louise Bourgeois makes it the occasion of a moral reflection, that, "if all those women who amuse themselves in debauching married men were formed in this way, the latter would soon withdraw from their *liasons*, to love their wives more than ever." Louise had reached the advanced age of seventy-two years, and without any thought of again figuring as an author, when she was persuaded by a publisher to issue a work called a "Collection of the Secrets of Louise Bourgeois." The chief revelation made by this publication was that it ought never to have appeared; and that any old woman who had reached the age of threescore and twelve could have written just as much to the purpose as the author of the Collection.

The means of instruction in midwifery were very limited in the time of Louise Bourgeois. Some women, as in a preceding age, studied with the regularly received matrons, whom they in turn succeeded. There was, besides, a school of obstetrics at the Hôtel-Dieu hospital, of Paris, which was superintended by a *sage-femme.* A list of those who have held this office from 1594 to 1775 is given by M. Rouyer, beginning with Jacqueline Fleury and ending with Madame Dugés. At a later date, theoretical courses of obstetrics, especially for sages-femmes, were established; thus, La Peyronie, first surgeon to Louis XV., created two chairs of midwifery, one of which was allotted for the instruction of midwives. In 1737, a school of midwifery was formed at the hospital of Strasburg, for the benefit of pupils of both sexes; but still, only a small number of females were enabled to profit by these and other means enlisted for the purpose. Of the latter, we may mention the diffusion of elementary knowledge in different parts of France by eminent female lecturers, duly authorized. First and most distinguished in this class was Madame de Bouisier Ducoudray, who was born at Clermont-Ferrand, in 1712. Madame Ducoudray, after many years of study, took her degrees at St. Come on the 26th of September, 1737 ; and by a decree of the police, on the 21st of February following, she received the title of sworn *sage-femme.*

Madame Ducoudray's first visit to the provinces to teach midwifery was at the invitation of M. de Tiers, a powerful seigneur in Auvergne, who busied himself not a little in the propagation of his vassals. Having seen the great loss of life among children and their mothers who perished in child-bed, he conceived the design of having educated midwives to prevent these disasters. With this view, he selected Madame Ducoudray, and sent for her, in order that she might instruct the more intelligent of the females on his estates. The teacher caused artificial pieces and manikins to be made for the purposes of demonstration, and she succeeded in forming tolerably skillful pupils. The noise of these proceedings reached the court, which issued a brevet authorizing the lady to teach the art of obstetrics and to indoctrinate pupils throughout the kingdom. Madame Ducoudray gave lectures in Paris and at many other towns, and prepared more than four thousand women to practice the art of midwifery. Ample room was there, indeed, for her instructions. On one occasion, in the country near Besançon, she was asked to see a poor peasant woman, exhausted by a long labor. This poor creature had been seated on a chair, with a log of wood placed under her thighs, so as to allow of any part of the protruding body of a living child to be cut off with a chopping-knife. A person crossing the floor would tread on the head and pieces of the limbs of this child, in order to extract the remainder of which, recourse was had to pot-hooks. At the moment of Madame Ducoudray's entrance the miserable patient breathed her last, exhausted by these brutal manœuvres. In other districts, the only process attempted was to put a cord round the neck of the child after the head had protruded, in order to aid the passage of the shoulders : often the head followed the cord in this traction, but without the rest of the body being expelled. In a part of Poitou visited by the lecturer, the whole science of the grannies in attendance on such occasions consisted in making the mother walk about as soon as the head had passed the *os externum*, so that the weight of the body of the child might complete the entire expulsion. We are afraid that examples of this gross ignorance of the first principles of obstetrics may still be found even at the present time in our own country.

Madame Ducoudray died in 1789. She was successful in procuring the founding of lying-in hospitals in several of the provincial towns. Her request for a similar establishment in Bordeaux was not acceded to. This good work was left to be carried out at the instance of her niece and pupil, Marguerite Guillomance, who afterward became Madame Coutanceau. The latter, in conjunction with her husband, had charge of the lying-in hospital at Bordeaux, which she was chiefly instrumental in founding. She wrote an elementary work, entitled "Summary Instructions, Theoretical and Practical, in Midwifery. For the Use of Midwives."

Madame Dugés was the last midwife-in-chief of the Hôtel-Dieu, of Paris. She was the daugher of dame Jonet, the sage-femme at the Chatelet, and the mother of Madame Lachapelle, and the grandmother of Professor Dugés. She gave striking evidences of her courage in the different epidemics with which the Hôtel-Dieu was assailed. The hospital was in those times in a sad state. Pregnant women and those in labor were received in a small and very unhealthy ward, in which they were crowded together; several women slept in the same bed, and such a crowding could not fail to give rise to destructive diseases. This deplorable state of things lasted until 1794. Not satisfied with even the amended hygienic state of the lying-in wards of the hospital, the authorities made choice of a more salubrious and better arranged establishment. Accordingly, the house known as that of Port Royal was selected, and converted into the lying-in hospital, (*Hospice de la Maternité.*) Madame Lachapelle, who had studied under the eyes of her mother, and seconded her in the discharge of her functions at the Hôtel-Dieu, was chosen to organize the new establishment. She succeeded her mother, Madame Dugés, in the chief superintendence of the hospital of the Maternité in 1797, on the death of the former. The husband of Madame Lachapelle was a surgeon attached to the hospital of St. Louis. She ultimately acquired a European reputation. In her teachings, she displayed method and precision, and had a simple and easy elocution. She omitted no means of instruction calculated to impress the minds of her hearers. In her practice she evinced great dexterity and address, and a readiness of adaptation to new and difficult circumstances of the cases of which she had the charge. Kind and gentle, she was known to all as "the good Madame Lachapelle." Her pupils were always sure of finding in her both a friend and an instructress, who was not only patient, but complaisant, while still retaining her authority and great influence. This estimable personage died at the comparatively early age of fifty years, of cancer of the stomach, the progress of which she concealed from her friends, so that she might continue the longer the discharge of her arduous duties. This skillful *sage-femme* has left behind her a very valuable and still prized work, "*The Practice of Midwifery*," in three volumes octavo, 1821–1825, the latter part of which was brought out under the supervision of her nephew, Professor Antoine Dugés.

Madame Lachapelle had a namesake, Mademoiselle Joanne-Louise Lachapelle, who was distinguished in the art of midwifery. She was carried off by the cholera in 1832, at the age of forty-three years. The lady first named, and whose professional life we have just sketched, was succeeded in the chief direction of the Maternité by Madame Legrand, who was obliged to withdraw from her post in 1839, on account of failing health. Her successor was Madame Charrier, who continued in the direction of the Maternité during a period of nineteen years, or until February, 1858, when she was carried off by apoplexy. The present incumbent, Madame Alliot, is spoken of in terms of commendation by M. Rouyer.

Among the deserved female celebrities in French obstetrics, the name of Mme. Boivin shines with the greatest lustre. She is quite a prolific writer. We have by her a memorial on obstetrics, a memoir on hydatic moles, another on the causes of abortion, on the pelvimeter, on the absorption of the placenta, etc. Finally, she has composed, in conjunction with Professor Dugés, a "Treatise on the Diseases of the Uterus and its Appendages," 2 vols., 8vo., with a colored atlas, in folio, 1833. She translated also different works in English and Latin; among others, Dr. Rigby's "Treatise on Uterine Hemorrhages," and a memoir on artificial delivery, and another on the Cæsarean operation, by Ferrario. Mme. Boivin was not long in gaining a European reputation: she was honored with numerous titles, received the degree of doctor of medicine in the University of Marbourg, and was made a member of the Society of Emulation, and of the Society of Practical Medicine in Paris.

Ample measures have been taken in France for the instruction of females who wish to practice midwifery. In the three Faculties there is annually a course of obstetrics, to which are admitted gratuitously this class of persons. In addition there is established in the most frequented hospital of each department an annual course of theoretical and practical midwifery, intended especially for the benefit of *sages-femmes* who attend without paying any fee. At the hospital of the Maternité there is a school of obstetrics destined to make midwives for all the departments of France. The female students may live two years at the Maternité, although they who at the end of the first year are prepared to undergo their examinations are allowed to present themselves for this trial. During their stay in the hospital they are required to follow a complete course of obstetrics, given by the surgeon of the hospital, a supplementary course by a house student (*un interne*) attached to the service of the women in child-bed. Besides this, they are called on to superintend ordinary labors, and to assist at those of a more complicated nature as they occur. In each clinical study they receive the aid of a *sage-femme* in chief.

We have not room for an account of the examinations to which the female candidates for a degree in midwifery are subjected. They consist of two series, and are adequately full and varied. The fees for their reception, as it is called, amount to 120 francs, or about 24 dollars.

Among English midwives we find the name of Elizabeth Nihell mentioned. She carried on an animated controversy with Smellie, and not long afterward published a "*Treatise on the Art of Midwifery, setting forth various abuses therein, especially as to the Practice with Instruments*," (London, 1760, 8vo.) This work was translated into French, and published under the following title, "*La Cause de l'Humanité deferée au tribunal de la Raison, ou Traité des Accouchements par les Femmes.*" A Mrs. Elizabeth Blackwell appeared as a writer in the early part of the last century. She attended Smellie's lectures, and engaged in the practice of midwifery, but was soon disgusted with it. Giving herself up to more congenial pursuits, she set about preparing a collection of notices of medicinal plants, under the title of "*Curous Herbal*," which, with 500 illustrative engravings, from drawings made by herself, was published in three volumes, in folio, (1736;) and a few years later in 2 volumes. It was translated into Latin, and published in Nuremberg in 1750 and 1760. Mrs. Blackwell was able, by the sale of her work, to procure the liberation from prison of her husband, who was a physician, and who had become involved by debts incurred in bringing out a work on agriculture. An Irish midwife, Mary Dunally, of Dungannon, who was born at the end of the eighteenth century, is known as a performer of the Cæsarean operation. The instrument which she employed on the occasion was a razor. After the extraction of the child she sent for a surgeon to dress the wound; during the two hours which elapsed before his arrival, she held the lips of the incision in close contact so as to prevent loss of blood. The woman survived the operation.

Among the German midwives, M. Rouyer mentions Justine Siegmundin and Ann Elizabeth Wiedmannin, who were the first to write treatises on obstetrics for the use of midwives.

Two women have acquired some celebrity for their skill in modeling in wax different parts of the human body. The first was Mademoiselle Bicheron, born in Paris about 1730. She showed an early passion for the study of anatomy, which, notwithstanding the restricted means of her parents, she gratified by purchasing books on the subject, and paying resurrectionists for bringing portions of dead bodies, which she dissected in her own chamber. Thus prepared by requisite anatomical knowledge, Mmdlle. Bicheron set about preparing models in wax, an occupation which she continued for thirty years. She visited London, where she was well received by John Hunter, and his pupil, Hewson. Her cabinet of anatomical preparations in wax was ultimately purchased by the Russian ambassador for the emperss Catherine II.

Still more famed was Anna Morandi, of Bologna, born in 1716, and who married, in 1768, John Manzolini, a somewhat celebrated anatomist of that period. Under her husband's tuition Anna learned drawing and anatomy; and, after having made the female genital organs her special study, she modeled in wax the uterus in a state of gestation, together with all the different positions which the fœtus could assume. A few years after the death of her husband, in 1785, saw this lady a member of several learned societies, including the Academy of Sciences of Bologna, and a professor of anatomy. Her lectures had a certain amount of vogue.

In drawing to a close our retrospect of women who have practiced medicine, and especially midwifery from the earliest times down to the present day, we have been influenced by what may seem to be due to the literature of the subject, but without, in any way, enlisting ourselves in advocacy of female efforts in this direction. Our own opinions, expressed in a previous number of this Journal, remain unchanged.

American women who have engaged in the practice of midwifery and general medicine will constitute the subject of a separate notice.

